# The Rise of Elite Short-Course Triathlon Re-Emphasises the Necessity to Transition Efficiently from Cycling to Running

**DOI:** 10.3390/sports7050099

**Published:** 2019-04-29

**Authors:** Joel A. Walsh

**Affiliations:** Illawarra Health and Medical Research Institute, University of Wollongong, Wollongong, NSW 2522, Australia; joelw@uow.edu.au; Tel.: +61-2-4298-1962

**Keywords:** cycle–run, transition, triathlon, performance, elite, training

## Abstract

Transitioning efficiently between cycling and running is considered an indication of overall performance, and as a result the cycle–run (C–R) transition is one of the most researched areas of triathlon. Previous studies have thoroughly investigated the impact of prior cycling on running performance. However, with the increasing number of short-course events and the inclusion of the mixed relay at the 2020 Tokyo Olympics, efficiently transitioning from cycle–run has been re-emphasised and with it, any potential limitations to running performance among elite triathletes. This short communication provides coaches and sports scientists a review of the literature detailing the negative effects of prior variable-cycling on running performance experienced among elite, short-course and Olympic distance triathletes; as well as discussing practical methods to minimise any negative impact of cycling on running performance. The current literature suggests that variable-cycling negatively effects running ability in at least some elite triathletes and that improving swimming performance, drafting during cycling and C–R training at race intensity could improve an athlete’s triathlon running performance. It is recommended that future research clearly define the performance level, competitive format of the experimental population and use protocols that are specific to the experimental population in order to improve the training and practical application of the research findings.

## 1. Introduction

Triathlon comprises several different racing formats (Table 1.) that can be generally categorised as short-course (super sprint/sprint); Olympic distance (short-distance/standard); or long-course (70.3/Ironman). Each category of triathlon places substantially different physical demands on the athletes [1], such as short-course triathlon (super-sprint/sprint distance) involving producing repetitive, high-intensity efforts due to the technical courses [2] changing the physiological demands of this type of triathlon [3], compared to the consistent, steady-state paced efforts required during long-course triathlon. However, all formats of triathlon require an athlete to transition from cycling-to-running. Subjective descriptions of perceived incoordination are commonly reported among triathletes of all levels during the cycle–run (C–R) transition [4], leading to a potential competitive advantage to athletes that can minimise the presence of impaired movement coordination during the C–R transition. Indeed, successful performance in triathlon is considered to be largely dependent on the ability of an athlete to overcome the specific physiological [5], neuromuscular [6] and biomechanical [7] complications associated with transitioning from cycling to running. Furthermore, recent evidence also suggests that specific effects of the C–R exist between elite male and female triathletes [8]. As a result of the recent increase in the number of short-course events held throughout the 2018/19 International Triathlon Union (ITU) World Triathlon Series (WTS), as well as the advent of the Super League Triathlon series (variable short-course distances), and the inclusion of the mixed relay event at the 2020 Tokyo Olympics, the relative importance of efficiently transitioning from C–R during triathlon, particularly during short-course formats, is re-emphasised and with it, any potential limitations to running performance. 

## 2. The Influence of Cycling on Running Performance in Elite Triathletes 

### 2.1. The Disparity Between Cycle–Run Testing Protocols and Race Demands 

Previous research has indicated that among highly-trained triathletes competing in short-course and Olympic-distance triathlon, any negative impact of prior cycling on running performance is minimal, compared to effects experienced by lesser trained, recreational triathletes [1,9,10,11]. However, a majority of research investigating the C–R use constant/steady state or incremental cycling protocols, prior to running. As a result, these findings may lack practical and training specificity for triathletes competing in draft-legal short-course and Olympic distance triathlon where cycling is highly variable with respect to both power output and cadence ranges [3] that would therefore, have a substantially different impact on running performance [2]. Subsequent studies have aimed to replicate the metabolic demand of cycling experienced during short-course triathlon, by prescribing constant cycling intensities based on a percentage of maximal aerobic power (~72% MAP) [12] or above the ventilatory threshold (~80% VO_2max_) [13]. Exercising at such intensities may reflect the average metabolic cost of the cycle leg of short-course and Olympic distance triathlon however, considering the variable nature of cycling during these formats of triathlon such testing protocols lack specificity, at least concerning elite draft-legal short-course and Olympic distance triathlon. As a result, it could be argued that non-specific testing protocols contribute to the lack of clarity regarding the understanding of the effects of prior cycling on running performance specific to elite short-course and Olympic distance triathlon. Alternatively, others [11] have used a run-cycle–run protocol to determine the differences between elite short-course versus elite long-course [12] and elite junior (male and female) versus elite senior triathletes [10,14]. While the findings of these experiments provide valuable information relating to identifying the physiological characteristics of select cohorts of elite triathletes, potentially aiding talent identification and monitoring training progression, they do not specifically outline potential changes among elite short-course and Olympic distance triathletes transitioning from C–R; that is a primary aim of this short communication. Therefore, discussions of past research in Section 2 and summarised in Table 2 of this short communication, will be limited to those articles that recruited elite triathletes and used a variable-cycling protocol [15] (i.e., variable power output and/or cadence) prior to running or a protocol that reflected the workload of a short-course triathlon [11] (Table 2).

### 2.2. The Effects of Variable-Cadence Cycling Protocols on Running Performance in Elite Triathletes

In order to specifically understand the impact of prior cycling on running performance previous researchers developed a protocol to determine the effects of cycling on the movement pattern of subsequent running (Table 2) [15]. These authors designed a moderate-intensity protocol aimed at minimising the impact of fatigue. In particular, they identified typical cadence ranges from data collected from elite triathletes competing at an international level to create their cycling protocol. Using this variable-cadence protocol (i.e., individually preferred cadence, 55–60, 75–80, and 95–100 rpm), on-going studies involving elite triathletes aimed to identify changes to the neuromuscular control [16], muscle recruitment patterns [17,18], kinematics [17,19], biomechanics [11] and economy [6,20] of subsequent running performance (Table 2).

Alternatively, others [21] have used moderate-intensity (variable-cadence) and high-intensity (power profile test) protocols to quantify changes to the neuromuscular control between an isolated/control run (IR) and C–R using electromyography (EMG) and joint angle waveforms (kinematics) sampled from the muscles of the lower limb (i.e., quadriceps, hamstrings and gastrocnemius and tibialis anterior) in elite triathletes (Table 2). Using the high-intensity, power profile test [21], these authors reported that overall, cycling minimally affects the stride-to-stride reproducibility of joint angles (<1.9°) or muscle recruitment patterns (<5.1%) during the early phase of subsequent running. Although, one participant did demonstrate altered muscle recruitment of biceps femoris following moderate-intensity, variable-cadence cycling, suggesting that adaptation to the C–R may be individual-specific. These results are in agreement with similar findings [6,17,19] indicating that most elite triathletes are able to effectively replicate pre-cycling running patterns when transitioning from moderate-intensity (variable-cadence) cycling. Moreover, using the same moderate-intensity, variable-cadence cycling protocol, no significant change in average muscle recruitment patterns or kinematics during the C–R, compared with an IR are evident [17,18]. However, among some elite triathletes (5/14 participants), muscle recruitment patterns recorded from the tibialis anterior muscle during the C–R closely reflect those recorded during cycling, and not the IR [17]. Alterations seen in some elite triathletes could be reflective of previous evidence that associates elite triathletes with a history of exercise-related leg pain (ERLP—5/10) with substantial (≥10%) alterations in EMG muscle patterns during C–R [19]. These authors suggested that those elite triathletes with a history of ERLP are ~2.4× more likely to have difficulty replicating neuromuscular control during running after variable-cadence cycling. Similarly, increased variability in muscle recruitment patterns recorded in the lower limb (coefficient of variation range = 18–37% has been observed during the early phase of C–R, i.e., 0–180 s), compared to IR [18]. These findings may suggest the presence of neuromuscular interference or the crossover of a generalised movement pattern that affects the control of running due to the prior, repetitive performance of the cycling movement pattern [15,16]. Furthermore, the changes in muscle recruitment patterns observed among some elite triathletes during the C–R, compared to an IR, have been associated with a reduction in running economy (3.7 ± 0.9%) [6]. Similarly, significant increases to the cost of running, respiratory exchange ratio, breathing frequency and heart rate have been reported among elite triathletes during the early phase of C–R, compared to an IR using the same moderate-intensity, variable-cadence cycling protocol (Table 2) [20]. These authors also reported an overall decrease in mean stride length (IR = 2.64 ± 0.18 v. C–R = 2.53 ± 0.17 m) and increase in mean stride frequency at the mean response time, ~63% of time to steady-state (IR = 85.7 ± 2.7 v. C–R = 87.0 ± 2.6 strides·min^−1^) and at 180 s (IR = 85.4 ± 2.6 v. C–R = 87.3 ± 2.7 strides·min^−1^) when running at the same self-selected velocity (13.5 ± 0.9 km·h^−1^) during the IR and C–R condition. Furthermore, changes in running mechanics suggest that leg stiffness increases in elite triathletes during running after a bout of fatiguing cycling [11]. Increased leg stiffness during C–R may indicate superior elastic energy storage and improved efficiency of repetitive stretch-shortening cycle movements during C–R [11]. Such biomechanical changes observed in elite triathletes, coupled with decreased stride length and increased stride rate, may act to counter-balance the potential negative changes to other physiological and neuromuscular variables in order to meet the demands of C–R.

### 2.3. The Effects of Variable-Power Cycling Protocols on Running Performance

The aforementioned research provides evidence to suggest that even at moderate-intensity variable-cadence cycling can have a negative effect on subsequent running performance in at least some elite triathletes. To date, no research has investigated the effects of variable-power cycling, at any intensity, on subsequent running in elite triathletes. However, results of a previous study suggest that variable-power cycling (i.e., 10–90 s intermittent efforts between 40–140% maximal aerobic power) had a greater negative impact on C–R performance, compared to constant-power cycling (i.e., 65% maximal aerobic power) among well-trained triathletes [22]. These authors reported significantly higher levels of blood lactate at the start of the C–R after variable-power cycling (64 ± 61%) compared to that after constant power cycling. The elevated blood lactate levels reflected a reduced running velocity at lactate threshold (~4 mM) of 0.6 ± 0.9 km·h^−1^ during the C–R following variable-power cycling. Furthermore, increased central and peripheral fatigue of knee extensor muscles after variable-power, compared to constant power cycling, have also been reported in well-trained triathletes [23]. The participants in this study did not complete any subsequent running after cycling however, the increased neuromuscular fatigue that reflected a reduced strength output of the knee extensors of 12.8 ± 6.1% that would likely contribute to a decrement in running performance. 

Overall, variable-cycling does not appear to heavily affect muscle recruitment patterns and joint kinematics in most elite triathletes competing in draft-legal short-course and Olympic distance triathlon, during the C–R period. Despite this, it is well acknowledged that the physiological cost of running during triathlon is substantially greater compared to IR [24], particularly during the early phase of C–R (0–180 s) [18]. Moreover, among those elite triathletes whose running performance is impacted by prior cycling, the effects are likely to have a substantially negative influence on overall performance; while increased variability of muscle recruitment patterns and altered stride mechanics during the early phase of C–R is evident among some elite triathletes and is more likely to be present in athletes with a history of ERLP.

## 3. Minimising the Effects of Cycling on Running Performance in Draft-Legal Short-Course and Olympic Distance Triathlon

### 3.1. The Role of Fatigue during Draft-Legal Short-Course and Olympic Distance Triathlon

Fatigue has been defined as “the failure to maintain force output, leading to a reduced performance” [25,26], “failure to maintain the required of expected power output” [27] and “any decline in muscle performance associated with muscle activity” [28]. Fatigue can be classified based on duration (acute/chronic), mental (cognitive or perceptual) and physical, referring to human motor performance [29,30]. A form of physical fatigue effecting performance in muscle fatigue that is considered the reduction in maximal force output and production of power in response to activity involving muscle contractions [31]. Muscle fatigue is often a primary factor in limiting athletic performance during high-intensity and/or prolonged exercise and is predicated by both central and peripheral mechanisms [30]. Peripheral fatigue involves changes at or beyond the neuromuscular junction independent of the central nervous system (CNS), involving mechanisms that impede or impair neuromuscular transmission, impulse propagation, calcium release and uptake in the sarcoplasm, substrate depletion (i.e., muscle glycogen depletion that precipitates fluid loss and increasing cardiovascular and metabolic strain), and contraction coupling [32,33]. Meanwhile, central fatigue occurs within the CNS resulting in a reduced neural drive to skeletal muscles [30,31] and is thought to be due to changes in serotonin, dopamine and noradrenaline (norepinephrine) in specific brain regions due to prolonged exercise [33]. It is important to note that the exact understandings of the mechanisms involved in central fatigue are still being debated. 

Every triathlete that has competed in elite-level draft-legal short-course and Olympic distance triathlon is likely to have experienced fatigue and as such, it is reasonable to suggest that increased fatigue, due to central and peripheral mechanisms, during elite draft-legal short-course and Olympic distance triathlon has a significant impact on running and overall performance. More specifically, the relatively duration and discipline distances of short-course and Olympic distance triathlon, relative to long-course formats, lends itself to higher intensity racing that exacerbates particular central and peripheral mechanisms responsible for fatigue. Indeed, the well reported increases in the energy cost, blood lactate concentrations, heart rate and oxygen uptake during the swim-cycle transition [34]; coupled with the documented increased oxygen cost [24,35,36,37], depletion of glycogen stores [24,35,36], muscle fatigue [24] and fluid loss [24,35,38], as well as loss of coordination [7,15], impairing neuromuscular control [6,17,18] and gait patterns [20,39] during the C–R transition is likely due to the complex build-up of central and peripheral fatigue mechanisms that significantly affects an elite triathlete’s physical performance, as evidenced by the progressive reduction in pace during short-course triathlon [40]. Therefore, it is necessary for elite triathletes, to limit the fatigue-related changes to physiological, neuromuscular or biomechanical variables that impact their performance by adopting strategies that attempt to minimise the build-up of fatigue and the accompanying negative impact on their performance [1,40]. Strategies (i.e., drafting, positioning and pacing) that would likely limit the fatigue-related changes to physiological, neuromuscular or biomechanical variables are discussed in the following section. Adopting such strategies would likely minimise effects of cycling on running performance during draft-legal short-course and Olympic distance triathlon, resulting in the conservation of energy that could translate in to an improved C–R transition and therefore, overall performance.

### 3.2. Impact of Drafting during Cycling on Running Performance

Typically, the energy expended in order to overcome rolling and frontal air resistance contributes to the increased cost of running after prior cycling during triathlon [1]. As such, the most effective means of minimising the effect of cycling on running performance is by drafting that involves riding behind one or several riders during cycling. Riding in a tightly formed group of riders (i.e., peloton/bunch) increases the drafting effect as the larger number of riders provides shelter from frontal air resistance (i.e., wind) [41]. Indeed minimising the wind resistance can lower the aerodynamic drag down to 50–70%, compared to riding isolated [42,43]. Moreover, sheltering in the mid-rear of a peloton can see drag reduced down to 5–10% [41], resulting in a substantially reduced energy expenditure [41,44]. In order to best maximise drag reduction when drafting, a rider can adopt a more aerodynamic road cycling position (i.e., riding in the drops) on the bike by decreasing their torso angle (i.e., between 0–16°) to reduce their frontal area [45] and therefore, aerodynamic drag by up to 20% [45,46]. Adopting a more aerodynamic cycling position does result in altered muscle activation patterns [47] and could potentially increase the physiological cost of cycling [48]. However, it appears that the benefits of maximising aerodynamics in order to improve the drafting effect outweigh any physiological cost [48]. Moreover, previous reviews have highlighted the benefits of drafting during triathlon that minimises the performance decrement during the C–R, contributing to a better overall race performance [1,2,7,40,49]. In particular, VO_2_ (~14%), ventilation (~30.8%), heart rate (~7.5%) and blood lactate concentrations are all reduced when drafting during 20 km of cycling at average speed of 39.5 km·h^−1^, compared to isolated cycling, resulting in a 4% improvement (running speed; 17.8 versus 17.1 km·h^−1^) in running performance during a sprint triathlon [50]. An even greater decrease in the energy cost of cycling has been shown when drafting behind a larger number of cyclists (eight versus, one, two and four cyclists) and has a significant influence on subsequent running performance [50].

### 3.3. The Importance of Positioning during Cycling on Running Performance

A triathlete can effectively improve their subsequent running performance by drafting to minimise energy expenditure, especially during the cycle leg of draft-legal, short-course and Olympic distance triathlon. However, the benefits of drafting are dependent on the number of athletes, their formation during cycling and the ability of the athlete to position and handle their bike within the bunch [40]. Indeed, it has been suggested that cycling speed increases substantially during the last kilometre of the cycle leg during elite triathlon due to athletes trying to attain a good position [7] toward the front of the bunch. Entering transition two (cycle–run: T2) at the front of the bunch would improve an athletes chance of minimising time in transition and may result in a better race performance [7]. Furthermore, the ability of a rider to maintain a good position at specific points during the race would likely result in conserving energy that could improve C–R performance. For example, riding at least 8 riders back from the front of the bunch during the early-latter parts of the cycle leg would maximise the drafting effect [50]. Additionally, moving in to the top 8 riders within the final kilometre(s) of cycling would reduce the intensity and number of accelerations performed [51] resulting in conservation of energy [2] and a better chance of moving through T2 more effectively than a rider positioned further back. Indeed, in road sprinting, positioning within the final kilometre(s) of a race (i.e., no less than 9 riders back) translates into a greater chance of winning, compared to a rider not positioned toward the front of the bunch [52]. As such, an athlete competing in draft-legal short-course and Olympic distance triathlon would benefit from having the skills necessary to navigate a bunch in order to effectively position themselves at certain points of the cycling leg to maximising the benefits of drafting in order to improve their running performance. Therefore, working on bunch riding and handling skills (i.e., cornering technique, rider-to-rider contact, effective braking, moving through gaps) to improve the athletes’ ability to maximise the drafting effect, while maintaining an optimal pedaling frequency and conservative pacing strategy, would be beneficial.

### 3.4. Effects of Pedalling Frequency during Cycling on Running Performance

Despite the reported energy saving benefits of drafting during elite short-course and Olympic distance triathlon [50], it is suggested that variability of power output and cadence increases as a by-product of drafting [53]. In particular, pedalling frequency (PF) influences the physiological cost and running performance during the C–R [13,54,55,56]. It has been reported that higher PF (cadence range 80–120 rpm) increases the oxygen cost and negatively affects subsequent running performance [55,56]. In order to combat the negative effects of high PF cycling and maximise running performance, it has been suggested that triathletes cycle at cadences between 70 and 80 rpm [1,57] and focus on reducing physical effort (power output) during the final minutes of the cycle leg [1]. However, a freely chosen cadence of ~90 rpm during submaximal cycling is considered biomechanically optimal [58,59]. Ideally, it is recommended that elite triathletes competing in draft-legal short-course and Olympic distance triathlon can minimise the effects of PF on C–R performance by maintaining a freely chosen PF close to ~90 rpm. Despite the understanding of the effects of PF on C–R performance, the underlying mechanisms of the physiological, biomechanical and neuromuscular adaptations are not thoroughly understood [2].

### 3.5. Effects of Pacing Strategies on Short-Course and Olympic Distance Triathlon Performance

The minimisation of energy expenditure through drafting and effective positioning during draft-legal short-course and Olympic distance triathlon could be complimented by adopting an efficient pacing strategy, that is a conscious plan to regulate physical effort [40], during the cycle leg. Furthermore, carrying a pacing strategy in to the subsequent run leg of a triathlon can substantially improve an athlete’s overall performance [5,60,61,62]. Triathletes of all levels, competing across most disciplines typically adopt a fast-start pacing strategy at the beginning of each new discipline (i.e., swim, cycle, run) during a race [40,61]. The fast-start strategy is thought to be due to high intensity of the swim leg in order to gain a good drafting positioning, “race dynamics” and environmental conditions [40]. However, the analysis of race pacing during elite ITU competition would indicate that a fast-start strategy is not the most efficient form of pacing [5,40,49,60,61,62,63]. However, differing pacing strategies may be required for certain disciplines of triathlon and between male and females triathletes [63]. Indeed, the current evidence suggests that top overall performers complete the first 400–500 m of the swim significantly faster than slower swimmers (i.e., positive pace strategy) [49]. This allows these athletes better positioning during the swim, among the top swimmers, maximising the drafting effect and resulting in a better swim exit that would lead to a more efficient cycle leg [40,61,62]. Alternatively, slower swimmers exit onto the bike further back and therefore, have to cycle faster, leading to an increased energy output that is inversely related to running performance [49,61]. However, it should be noted that previous research suggests that reducing swim speed (80–85% of mean swim speed) resulted in faster cycling performance [64]. Reducing swim speed during a race-situation may be detrimental to overall performance therefore, improving swimming ability that enables a triathlete to start-fast during the first 400–500 m and then maintain a steady swim pace below 90% of their maximal speed [40,64] could be seen as a preferred pacing strategy. 

As previously mentioned, cycle pacing during draft-legal triathlon is heavily dependent on the number of riders in the pack, its configuration and the tactical location of other athletes [40]. Additionally, the importance of maintaining a position at the front of the bunch in an attempt to maximise the drafting effect and the pace of the leading athlete influence the pacing strategy of the cycle discipline [40]. As such, a fast-start pacing strategy is most commonly seen among all respective bunches, however, the pace of the “chasing” bunches likely remains higher than that of the front/lead bunch who often reduced their speed in the final few kilometres prior to the C–R [40]. Together, these “race dynamics” often results in variable-paced cycling. 

Similar, to the swim and cycle disciplines, triathletes commonly adopt a reverse J-shaped pace during triathlon running [40,49,61], characterised by a fast-start that declines through the mid-race and then ramps during the final phase [40]. It is unknown whether this run pacing strategy is the most effective for overall performance; rather it is likely determined by “race dynamics”. The reverse J-shaped pacing strategy contradicts the current evidence that advocates for an even-paced run strategy [5,60,61]. Specifically, adopting a running speed 5% slower than an athlete’s average 10 km pace, during the first kilometre of the run, resulted in a superior run performance [5]. These authors suggested that the slower pace during the early C–R period reduced the development of fatigue, compared to a fast-start strategy, contributing the improved running performance. However, during short-course (i.e., sprint distance, super sprint, and mixed relay) a fast-start strategy may be more beneficial [40,65,66]. A fast-start pacing strategy has been shown to improve oxygen uptake kinetics during exercise lasting between 3–7 min, indicated by improve 3-min cycling performance [66]. 

Based on the current evidence the following pacing strategies for draft-legal short-course and Olympic distance triathlon are considered most efficient in order to achieve a high overall placing:

(a) Swim: fast 400–500 m, followed by adopting an even pace, below ~90% maximal swim speed, for the remainder of the swim.
(b) Cycle: despite the likelihood of a variable-paced cycle, athletes should aim to maximise the drafting effect through adopting an aerodynamic positioning and ensuring they are positioned efficiently at specific points during the cycle leg. Additionally, maintaining a PF of ~90 rpm and a constant pacing strategy, as well as decreasing their efforts during the latter phase (at least during the final 1 km) of the cycle discipline in order to conserve energy for the C–R. Alternatively, a fast-start strategy is recommended based on the previously reported superior performance during short duration cycling exercise.
(c) Run: athletes should aim to adopt a slightly slower pace (~5% below 10 km pace) during the first kilometre during the C–R and hold a constant pace throughout the run discipline. Alternatively, during short-course triathlon (i.e., sprint distance) a fast-start strategy is recommended.

It is acknowledged that the aforementioned pacing strategies are heavily dependent on other variables and as such athletes will need to react to specific race situations accordingly. However, the pacing strategies provided are based on data collected during elite draft-legal Olympic distance triathlon, namely the 2002 ITU Lausanne World Cup [61], 2007 ITU Beijing World Cup [63,67] and the 2009 European Triathlon Championships [60], and as such can be considered specific to draft-legal short-course and Olympic distance triathlon. 

### 3.6. Effect of Swimming on Cycling Performance Prior to Running

Transitioning from swimming, a predominantly upper-body movement, to cycling that is a predominantly lower-body movement presents some difficulties, mainly blood pooling in the arms [2] that may delay redistribution of blood flow around the body when moving from a supine to upright posture. Moreover, it has been demonstrated that high-intensity swimming substantially increases the physiological cost during subsequent cycling [40,68]. In particular, reduced efficiency (~13%), increased [La-] (~56%) and elevated VO_2_ (~5%) have been reported during the first 5-min of a 30-min bout of cycling [34]. However, in comparison to the C–R transition, it is reasonable to suggest that there is a relatively small amount of research investigating the effects of swimming on subsequent cycling and running performance. Apart from the difficulties of reliably conducting swim-based laboratory testing, the lack of focus on the swim-cycle transition, compared to the C–R, is likely due to the suggested weak correlation (r = 0.9730) between swim duration (~10% of total race time) and overall race time [69]. These authors suggested that run performance is considered a better overall predictor of triathlon performance (r = 0.97) and therefore, improving the C–R performance would likely contribute to a better race performance. However, the correlation between the time of the discipline (i.e., swim, cycle or run) may not be entirely reflective of performance, especially at an elite level [68]. Analysis of race data has shown that swimming performance has a substantial impact on an athlete’s overall performance, particularly during draft-legal sprint and Olympic distance triathlon [1,49]. Analysing race data from an ITU World Cup event it was reported that the races’ top performers swam significantly faster during the first 400–500 m putting them in to a better swim-exit position [49]. Consequently, the slower swimmers had to cycle significantly faster during the first 20 km of the cycle leg, likely compounding the negative effects of the C–R. These authors concluded that running performance largely determines overall performance and is inversely related to cycling speed in the early period of the cycle leg. These findings indicate that a superior swim performance can largely reduce the energy cost of cycling, contributing to an improved running performance. However, continued research by these authors demonstrates that pacing strategies during triathlon is different between sexes [61]. Interestingly, using similar ITU World Cup race data, these authors concluded that cycling performance is more important for elite female triathletes, compared to their male counterparts. It was reported that male triathletes who swam slower had to ride significantly faster in order to ‘bridge’ to the front group(s) prior to the C–R transition. Furthermore, those athletes in the first pack (superior swimmers) did, on average, run faster during the run leg compared to the athletes in packs two and three (slower swimmers). Alternatively, it has been proposed that elite female triathletes with superior swim and cycle performances significantly improve their chances of a higher overall finishing position compared to those athletes with a weaker swim/bike capacity [61]. Such findings have substantial training and race strategy implications in those elite female athletes with a superior swim/bike performance could isolate their competitors who possess a superior run performance but who are weaker swim/bikers. Overall, this race data indicates that superior swimming performance can substantially minimise the decrement in running performance during triathlon in both elite male and female athletes. 

### 3.7. Specific Training Aimed at Minimising the Potential Effects of Cycling on Running Performance

Replicating race situations in training is a difficult task. However, for those elite triathletes whose running performance is affected by prior cycling, specific training aimed at adapting to the C–R transition should be considered. Common training modalities of elite triathletes has been previously reported however, the data suggests that C–R type sessions are not regularly used [70]. Furthermore, to the author’s knowledge, only one previous study has looked at the effects of repetitive C–R training on performance [71]. These authors reported that six weeks of multicycle–run training sessions did not improve C–R performance however; improvements in the C–R transition were evident among the triathlete cohort. Despite the lack of evidence regarding C–R training in elite triathletes, such training may be beneficial for those elite triathletes identified as having difficulty replicating muscle recruitment patterns and neuromuscular control of running after cycling. This type of sequential “brick” training may serve to evoke a training effect that refines the central nervous system’s use of a generalised movement strategy [72] that may govern cycling and running motor patterns among elite triathletes sensitive to the negative effects of the C–R [16]. Furthermore, the current literature suggests that variable-cycling does increase the physiological cost (i.e., [La-], R_E_, C_R_, RER and HR) of subsequent running in elite short-course and Olympic distance triathletes [11,20,73]. Incorporating multicycle–run training sessions in to the training programs of elite triathletes may assist with developing physiological adaptations to the C–R [71]. However, our current understanding of the training effects of back-to-back, “brick” or multicycle–run sessions on adaptation to the C–R are limited and requires further investigation. 

However, should coaches and sports scientists use C–R training sessions as part of an elite short-course and Olympic distance triathlete’s training program, the intensity should be reflective of the demands experienced during a race and completed as such. Currently, moderate-intensity variable-cycling appears sufficient enough to induce effects experienced during the C–R; however, to replicate race-specific demands during training, variable and repetitive high-intensity C–R efforts would likely be more beneficial [71]. The implementation of C–R training sessions that use variable-power/cadence cycling prior to running is likely to evoke a more specific training stimulus that may contribute to an improve ability to absorb repeated, high-intensity accelerations during draft-legal triathlon [74] and therefore, minimise any negative effect during the C–R period leading to a better overall performance. In addition, adopting a reverse J-shaped paced running strategy (i.e., fast-start, gradual decrease in speed, with a late acceleration of speed), commonly experienced during Olympic distance triathlon [40], would further exposure select elite triathletes to race-specific demands of the C–R transition and potentially improve adaptation. However, it should be noted that alternate pacing strategies are considered to be more efficient and may result in better overall performance. Another means of evoking the specific characteristics of the variable nature of cycling and running experienced during elite short-course and Olympic distance triathlon would include training with other elite triathletes where the pace of cycling and/or running is influenced by the individuals [40]. 

## 4. Future Recommendations 

### 4.1. Considerations for Future Research

Currently, there is a limited amount of research investigating the effects of variable-cycling on running performance among elite triathletes and as a result, there is considerable scope to conduct further research within this area. However, based on the lack of clarity within the literature, it is recommended that future research should:

(I) More rigorously define the performance/ability level of the experimental population based on pre-defined criteria. For example, elite triathletes can be defined using a previously defined criteria [75] or based on an ITU world ranking inside the top 125 [2] or their current competitive level, i.e., national/international level (Table 2). Alternatively, using physiological characteristics, such as VO_2max_ or VO_2peak_, to define the “trained” status of the triathlete population [76,77] may minimise the miscategorisation of the experimental cohort and therefore, improve the training implications of the research, specific to the target triathlete population.
(II) Define the format of triathlon that the testing populations competes. As previously outlined in this article (Table 1), there are various formats of triathlon that can be categorised as short-course, Olympic or long-course, each requiring differing skill sets and physiological output of triathletes. Defining the racing format of the testing population would largely improve the translation of the research findings.
(III) Implement testing protocols that are specific to the category of triathlon (i.e., short-course, Olympic or long-course) in which the testing population competes. For example, if the testing population predominantly competes in short-course triathlon, characterised by variable-, high-intensity cycling, the testing protocols should reflect this. Such testing protocols can be developed or refined using previously reported race data [49,61,78]. Although, within the current literature two field-based [79,80] and three laboratory-based [11,15,81] testing protocols have been reliably validated [15,82]. Therefore, should researchers not use any of the aforementioned protocols, they should at least adopt a testing protocol that resembles the changes a triathlete, male or female, elite or novice, is likely to experience.
(IV) Despite the previously mentioned changes in running performance after submaximal variable-cycling, experimental testing should be conducted using intensities that better reflect the demands that the triathlete cohort are likely to experience during racing.
(V) Investigate the use of alternative methodological techniques to help quantify the mechanisms influencing C–R performance. For example:
  - Techniques including evoking compound motor action potential (M_max_) in peripheral nerves (i.e., peripheral nerve stimulation), along with the use of transcranial magnetic stimulation and electrical stimulation can provide an understanding of the level of neuromuscular fatigue experienced during exercise [83,84]. In particular, these techniques have been used to detect changes in motoneuron excitability of the quadriceps muscles during exercise [84,85] and could be used to analyse the effect of the C–R on motoneuron excitability of the leg musculature as a way of identifying any potential neuromuscular fatigue.
  - Previously, intra-individual variability of gait cycles and cycling patterns have been analysed using variance ratio formulae [86,87]. Such a formula could be used to analyse the reliability and consistency of replicating running gait patterns in order to quantify the reproducibility of efficient muscle activation patterns during C–R performance. For example, plotting muscle activation pattern across a time series during the C–R period would provide a visual representation of any athlete that has difficulty reproducing pre-cycling running patterns.

### 4.2. Incorporating New Technologies and Techniques into Triathlon Research

The increasing use of technology (i.e., power meters, GPS, blood lactate analysers, digital training platforms) in triathlon provides researchers with the ability to collect and analyse in-field data specific to the demands of each discipline. Access to such data could assist in the development of more specific laboratory-based experimental protocols and the refinement of analysis procedures that would carry a greater degree of practicality for the target triathlete populations. In particular, the use of power meters during cycling provides coaches and sports scientists with detailed power profiles of race course that can be used to develop specific training sessions that can be implemented during race-specific preparation. Meanwhile, the use of wearable GPS tracking units could be used to collate training load, paired with online training platforms, to minimise occurrence of soft tissue injuries [88,89,90]. Furthermore, the paired use of power meters and GPS can be used by the athlete and coaching staff to develop pacing strategies for specific races, as well as allowing the athlete to monitor their pacing throughout an event in order to maximise their overall performance. Additionally, other wearable motion analysis devices based for example on inertial measurement units (e.g., Leomo^®^ TYPE-R, Leomo, Boulder, CO, USA) could be used to provide real-time data on power output, kinematics and biomechanical alterations outside of a controlled laboratory setting. Using such “live” and in situ motion analysis would likely improve the ability of coaches and researchers to more readily identify those triathletes susceptible to negative C–R performance and provide on-the-spot feedback to athletes that would provide considerable advancement in the specificity of training for triathlon. Additionally, this technology could be used “live” at races or as part of testing procedures in order to provide athletes with direct feedback of the physiological, kinematic and biomechanical changes they are experiencing during the C–R, and therefore, potentially assisting with them adopting different pacing strategies and/or adjusting their race tactics accordingly.

Investigating more sensitive measures of physiological, neuromuscular and biomechanical alterations surrounding the C–R transition should also be encouraged. For example, sagittal plane kinematic analysis suggests elite triathletes are able to replicate pre-cycling running patterns during the C–R transition [16]. However, analysing motor patterns using two-dimensional sagittal plane kinematics is typically limited to quantifying the excursion of individual joint angles and is limited in its ability to account for temporal changes or changes to the coordination of the segments of the leg during a gait cycle. Alternatively, analysing the coordination of the segments of the leg (i.e., thigh, shank and foot) together, using intersegmental coordination, has suggested that neural control of the leg is highly regulated in order to maintain equilibrium during walking and running [91,92,93]. Furthermore, the regulation of segmental coordination of the leg, during locomotion, has been linked to movement economy [91,92]. Applying such a measure may assist in the identification of those elite triathletes susceptible to kinematic and physiological changes induce during C–R performance.

## 5. Conclusions

In conclusion, the current evidence available shows that variable-cycling has minimal effect of running performance in elite triathletes. However, cycling does negatively affect running performance in some elite triathletes. Specifically, reductions in running economy, increased variation in muscle activation patterns and changes to stride patterns at intensities below that experienced during racing have been reported among affected elite triathletes. Furthermore, it is reasonable to estimate that these negative effects would be amplified under high-intensity conditions [16]. Meanwhile, pacing strategies employed during the swim and cycle disciplines appear to influence subsequent running and overall performance among all elite triathletes. Despite this, there are ways that an athlete and their coach can minimise the physiological, neuromuscular and biomechanical decrements associated with prior swimming and cycling on C–R performance, such as through drafting, effective bunch positioning, adopting efficient pacing strategies, pedaling frequency and C–R training. It is likely that short-course triathlon racing will continue to gain momentum as a specialty discipline within triathlon, similar to that of 70.3 and Ironman. In response, future research should apply specific criterion for defining the caliber of the experimental cohort in order to not miscategorise the ability of the athletes and use testing protocols that reflects the demands of racing experienced by the experimental population. Continued research should aim at refining and developing new laboratory analysis procedures and in-field testing methods that improve the ability of coaches and scientists to identify those elite triathletes susceptible to the negative effects of cycling on running performance, in order to improve specific training strategies. Additionally, a continued focus on the analysis of current race data will provide a more in-depth understanding of the ever-changing demands of draft-legal short-course and Olympic distance triathlon that will in-turn shape the direction of future research. 

## Figures and Tables

**Table 1:** Commonly raced formats of triathlon.

Event	Swim	Bike	Run	Course Structure	Event Characteristics
Super sprint ^†^	400 m (0.25 mi)	10 km (6.2 mi)	2.5 km (1.6 mi)	Short circuit racing, highly technical	Repetitive, high-intensity accelerations, high power/speed, technical courses, highly tactical, drafting/non-drafting, emphasis on C–R transition.
Sprint ^†^	750 m (0.47 mi)	20 km (12 mi)	5 km (3.1 mi)	Circuit racing, criterium-style bike leg, relatively technical	Repetitive, high-intensity accelerations, high power/speed, technical courses, highly tactical, drafting/non-drafting, emphasis on C–R transition.
Olympic *	1.5 km (0.93 mi)	40 km (25 mi)	10 km (6.2 mi)	Often circuit racing, draft/non drafting bike leg, some technical aspects	Repetitive, high-intensity accelerations, high power/speed, technical courses, highly tactical, drafting/non-drafting, emphasis on C–R transition energy conservation/minimising physical effort.
70.3 ^§^	1.9 km (1.2 mi)	90 km (56 mi)	21.1 km (12 mi)	Long course, non-drafting bike leg, out-and-back courses, non-technical	Prolonged, submaximal steady-state efforts, management of energy consumption and effort, non-drafting cycle leg, non-technical course.
Ironman ^§^	3.9 km (2.4 mi)	180 km (112 mi)	42.2 km (26.2 mi)	Long course, non-drafting bike leg, out-and-back courses, non-technical	Prolonged, submaximal steady-state efforts, management of energy consumption and effort, non-drafting cycle leg, non-technical course.
Mixed relay *^,†^	300 m (0.19 mi)	8 km (5.0 mi)	2 km (1.2 mi)	Short circuit racing, highly technical, similar to super sprint events	Repetitive, high-intensity accelerations, high power/speed, technical courses, highly tactical, drafting/non-drafting, emphasis on C–R transition.

* denotes Tokyo 2020 event; ^†^ denotes short-course event; ^§^ denotes long-course event.

**Table 2:** Physiological, neuromuscular and biomechanical effects of prior cycling on running performance specific to elite short-course and Olympic distance triathletes.

Participants	Protocol	Effects	Conclusions	Reference
8 elite triathletes (1 male) —international level (top 50 world ranking)	Run–cycle–run - 7-min run at sprint distance race-pace (18 and 15.1 ± 0.6 km·h^−1^) - maximal incremental cycle (70 W increments/3-min from 70–280 W, 35 W increments/2-min to volitional exhaustion) - 7-min run at sprint distance race-pace (18 and 15.1 ± 0.6 km·h^−1^)	- ↑ [La-] between 1st and 2nd 7-min run - mean 3.7% ↓ C_R_ during 2nd 7-min run v. 1st 7-min run - mean 4.2% ↓ ΔH_STRIKE_ during 2nd 7-min run v. 1st 7-min run. - Small mean (4.3%) Δ C_M_ during 2nd 7-min run v. 1st 7-min run.	- Cost of running is not significantly affected by a fatiguing bout of cycling in elite triathletes, despite changes in [La-] between 7-min run bouts. - reduced mechanical changes during the 2nd 7-min run suggest that leg stiffness is better preserved in elite triathletes.	Millet, Millet, Hofmann and Candau (2000)
8 elite triathletes (1 male) —international level (top 50 world ranking)	see Millet et al. (2000)	- No significant Δ the mechanical or kinetic cost of running pre- and post-fatiguing cycling	- A prior bout of high- intensity, fatiguing cycling does not affect the subsequent running mechanics in elite triathletes.	Millet, Millet and Candau (2001)
16 elite triathletes —national/international level	see Chapman et al. (2009) - 10-min CR - 20-min variable-cadence cycling followed by a 30-min TR	- No Δ TA EMG patterns CR v TR - No Δ SL, SD or kinematic joint angles CR v TR - 5/14 did show ↓ EMG amplitude of TA during TR	- Short periods of variable-cadence, moderate-intensity cycling does not affect running kinematics or SL among elite triathletes. - However, cycling may influence muscle activation patterns during TR in some elite triathletes.	Chapman, Vicenzino, Blanch, Dowlan and Hodges (2008)
34 elite/highly-trained triathletes —national/international level —World championship qualified —Olympic distance specialisation	see Chapman et al. (2009) - 10-min CR - 20-min variable-cadence cycling followed by a 30-min TR	- No Δ joint kinematics or EMG muscle patterns in most triathletes (70%) - 30% of triathletes showed Δ EMG patterns during TR - Δ EMG muscle patterns associated with 3.7 ± 0.9% ↓ R_E_ (↑ VO_2_)	Prior variable-cadence cycling impairs neuromuscular control on some elite triathletes that are associated with reduced TR economy	Chapman, Vicenzino, Hodges, Dowlan, Hahn, Alexander and Milner (2009)
34 elite/highly-trained triathletes —national/international level —World championship experience —Olympic distance specialisation	see Chapman et al. (2009) - 10-min CR - 20-min variable-cadence cycling followed by a 30-min TR	- No Δ joint kinematics - EMG patterns differed by ≥10% between CR and TR in 5/24 control triathletes and 5/10 triathletes with a history of ERLP	Potential association between ERLP and neuromuscular control during TR in elite triathletes with a history of ERLP	Chapman, Hodges, Briggs, Stapley and Vicenzino (2010)
7 elite triathletes (3 female) —international level —national representatives at world level	Low-intensity - see Chapman et al. (2009) High-intensity cycling - see Quod et al. (2010)	- No Δ R_E_ or neuromuscular control of the left leg during TR following low and high intensity cycling. - No Δ lower limb kinematics - No Δ EMG patterns following high-intensity cycling - 1/7 triathletes showed altered EMG patterns during TR following low-intensity cycling	- Low and high intensity variable cycling does not adversely impact TR neuromuscular control of R_E_ in elite triathletes	Bonacci, Saunders, Alexander, Blanch and Vicenzino (2011)
6 triathletes —National/international level —ITU Olympic distance race experience	see Chapman et al. (2009)	- No mean Δ lower limb EMG muscle activity patterns between CR and TR - ↑ variability of EMG activity during TR	- lower limb EMG activity patterns are not substantially influenced by variable-cadence cycling in elite triathletes	Walsh, Stamenkovic, Lepers, Peoples and Stapley (2015)
8 triathletes —National/international level —ITU Olympic distance race experience	see Chapman et al. (2009)	- ↑ C_R_, RER and HR at MRT and 10th minute of TR v CR - ↓ SL, ↑ SR during TR v CR	- moderate-intensity variable-cadence cycling significantly affects physiological and stride pattern variables during TR, compared to CR.	Walsh, Dawber, Lepers, Brown and Stapley (2017)

Key: [La-] blood lactate concentration; C_R_ energy cost of running; C_M_ mechanical cost; ΔH_STRIKE_ vertical displacement of centre of mass during braking phase; CR control run; TR transition run; Δ change in; TA tibialis anterior; EMG electromyography; SL stride length; SD stride duration; ↓ decrease; ↑ increase; W watts; R_E_ running economy; RER respiratory exchange ratio; HR heart rate; VO_2_ oxygen uptake; ERLP exercise-related leg pain.
